# Manipulation of surface charges of oil droplets and carbonate rocks to improve oil recovery

**DOI:** 10.1038/s41598-021-93920-3

**Published:** 2021-07-15

**Authors:** Jian Hou, Ming Han, Jinxun Wang

**Affiliations:** 1 Aramco Asia Beijing Research Center, Beijing, China; 2 EXPEC Advanced Research Center, Saudi Aramco, Saudi Arabia

**Keywords:** Energy, Surface chemistry

## Abstract

This work investigates the effect of the surface charges of oil droplets and carbonate rocks in brine and in surfactant solutions on oil production. The influences of the cations in brine and the surfactant types on the zeta-potentials of both oil droplets and carbonate rock particles are studied. It is found that the addition of anionic and cationic surfactants in brine result in both negative or positive zeta-potentials of rock particles and oil droplets respectively, while the zwitterionic surfactant induces a positive charge on rock particles and a negative charge on oil droplets. Micromodels with a CaCO_3_ nanocrystal layer coated on the flow channels were used in the oil displacement tests. The results show that when the oil-water interfacial tension (IFT) was at 10^−1^ mN/m, the injection of an anionic surfactant (SDS-R1) solution achieved 21.0% incremental oil recovery, higher than the 12.6% increment by the injection of a zwitterionic surfactant (SB-A2) solution. When the IFT was lowered to 10^−3^ mM/m, the injection of anionic/non-ionic surfactant SMAN-l1 solution with higher absolute zeta potential value (ζ_oil _+ ζ_rock_) of 34 mV has achieved higher incremental oil recovery (39.4%) than the application of an anionic/cationic surfactant SMAC-l1 solution with a lower absolute zeta-potential value of 22 mV (30.6%). This indicates that the same charge of rocks and oil droplets improves the transportation of charged oil/water emulsion in the porous media. This work reveals that the surface charge in surfactant flooding plays an important role in addition to the oil/water interfacial tension reduction and the rock wettability alteration.

## Introduction

Surface charge is an important feature of both emulsions and solid particles in electrolyte solutions, which has a significant effect on chemical and physical properties, such as the stability of the emulsions, and the adsorption of ionic species on the solid particle surface. In the oil and gas industry, a large amount of studies focused on the surface charges of rock and crude oil, and the effects on the liquid/liquid and liquid/solid interactions, for the improvement of hydrocarbon production in clastic and carbonate reservoirs^1^. As the surface charge is hard to be determined directly, zeta potential measurements are most commonly applied to quantify the electrostatic properties of carbonate rock surfaces and oil droplets in solutions. The quantitative changes in the magnitude of surface charge can result in correspondent changes in the zeta potentials^2,3^. It was reported that the zeta-potential of carbonate minerals was reported to show different trend from the clastic minerals in low salinity brines^4^. It is worth to note that the carbonate reservoir is composed of calcite or dolomite while the clastic reservoir, typically sandstone reservoir, is mostly composed of quartz. In this work, the study focuses on the surface charge factor on oil production in carbonate reservoirs.

Emulsions composed of crude oil in salty water usually show a negative surface charge or negative zeta-potential for two main reasons: (1) the dissociated fatty acid of crude oil is adsorbed on the oil/water interface^5–8^; (2) the negatively charged ions in solutions, such as OH^−^ or Cl^−^, interact with the nonpolar component of crude oil and make the zeta-potential negative^9^. On the contrary, the carbonate surface usually carries positively charge in reservoir condition^10^. The zeta-potential of carbonate rock particles is influenced by pH, ionic strength, and especially by the potential determining ions in the electrolyte solutions as a result of complicated calcite surface reactions^11–16^.

Efforts in investigating the interaction of charged oil droplets and rock surfaces are made in controlled salinity water injection technique^17–19^. It was reported that when the oil droplets and carbonate rock surfaces were the same charged, the wettability alteration from oil-wet to more water-wet condition in turn releases the oil attached on the rock surfaces and improves oil production. On the contrary, the oppositely charged oil droplets and rock surfaces leads to an oil-wet state and impede the oil production in the controlled salinity waterflooding^15,20^.

In a surfactant flooding process, the mobilization of residual oil by surfactant is usually attributed to the oil/water interfacial tension reduction and/or the wettability alteration^21,22^. Zeta-potential measurements were usually conducted for investigating the adsorption induced wettability alteration and the stability of oil/water emulsions^23^. For example, the wettability of rock in surfactant solutions is closely related to the adsorption orientation of the surfactant. Head-out adsorption is preferred to be water-wet, and tail-out adsorption is preferred to be oil-wet. This orientation is determined by the surface charge of rock, the ionic type and the structure of surfactants^24^. When oil droplets are stabilized by surfactants, emulsions show high zeta-potential (negative or positive) to be stable. Emulsions with low zeta-potentials tend to coagulate or flocculate^25–27^.

However, the effects on oil production of the surfactant induced interactions between the charged oil and rock were not fully discussed. This work aims to investigate the zeta-potentials of oil droplets and rock particles in anionic, cationic, zwitterionic and non-ionic surfactants, and demonstrate the oil mobilization potential of these surfactants by micromodel displacement tests. This study provides further insight into the oil recovery mechanisms of surfactant flooding in addition to the IFT reduction and wettability alteration.

## Results

### Zeta-potentials of oil droplets

**Figure 1:**
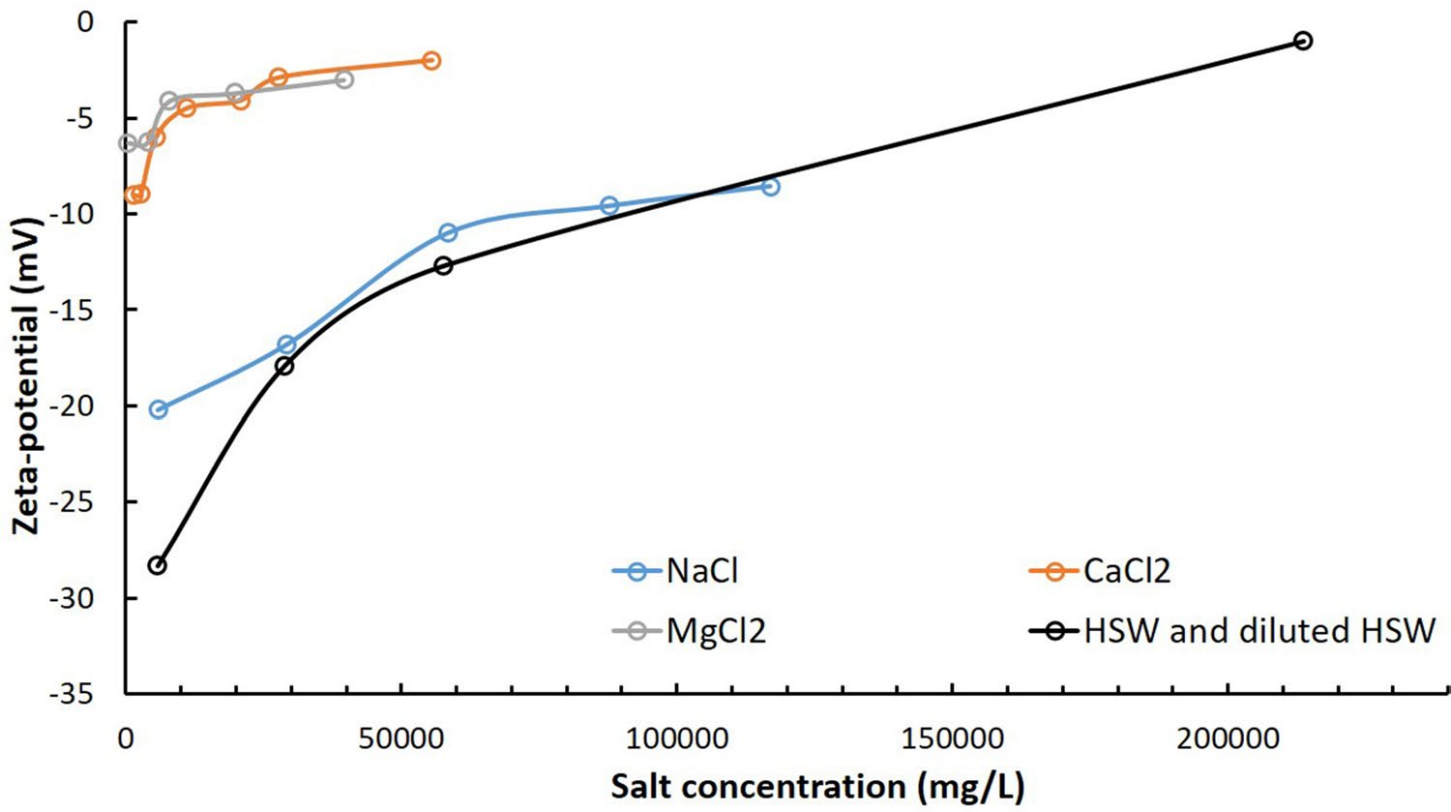
Zeta-potential of oil droplets in different salt solutions.

The effects of Na^+^, Ca^2+^ and a high salinity water of mixed ion species on the zeta-potential of oil droplets were discussed in the first place. The zeta-potentials of oil in five different NaCl concentrations, 0.1 mol/L (5850 mg/L), 0.5 mol/L (29,250 mg/L), 1 mol/L (58,500 mg/L), 1.5 mol/L (87,750 mg/L) and 2 mol/L (117,000 mg/L), were measured, and the results are presented in Fig. 1 (blue line). It shows that the zeta-potential increases with the increasing Na^+^ concentration to about − 9 mV in the tested range. This is in consistent with the phenomenon that the zeta-potential of colloid at high salt concentration as a finite value^2^. The increase of zeta-potential in Na^+^ solution is mainly due to the suppression of the double electric layer as the increase of ionic strength in the solution. For the zeta-potential study of oil droplets in Ca^2+^ solutions with CaCl_2_ concentration from 1388 to 55,500 mg/L (Ca^2+^ from 500 to 20,000 mg/L), it shows a much faster increase of zeta-potential at low Ca^2+^ concentration and then the increase rate slows down (Fig. 1 orange line). With the increase of Ca^2+^ concentration, the zeta-potential is stable at the value of around − 2 mV. The result demonstrates that the calcium ions have a stronger interaction with oil than sodium ions other than the suppression of the double electric layer. The microscopy image of emulsions in CaCl_2_ presents bridging effect that the Ca^2+^ would attract the negatively-charged oil emulsions and bridge them into larger emulsions solutions (Figure S1, Supplementary Information). In the investigation of the zeta-potential of oil droplets in MgCl_2_ solutions in the range of 396–39,584 mg/L (Mg from 100 to 10,000 mg/L), the zeta-potential of oil droplets presents similar trend as the addition of Ca^2+^ (Fig. 1 gray line). In the case of zeta-potential of oil droplets in mixed ion brines like high salinity water (HSW), diluted HSW and connate water in black line (connate water composition referred to Han et al.^28^), it shows a synergetic effect of different ions.

In surfactant flooding process, the zeta-potential of oil/water emulsion is affected by both the water salinity and the surfactant molecules at the same time. The surfactants can partition at the oil/water interface through the interaction between the hydrophobic alkyl chain and the non-polar components of crude oil, which affects the surface charge and zeta-potential of oil droplets in solutions. The zeta-potential of oil droplets in four differently charged surfactants SDS-R1, DTAB-R2, OP-A1 and SB-A2, as well as two surfactant mixtures, were investigated. The chemical structures of the surfactants and their critical micelle concentrations in HSW are provided in Figure S2 and Table S1.

**Figure 2:**
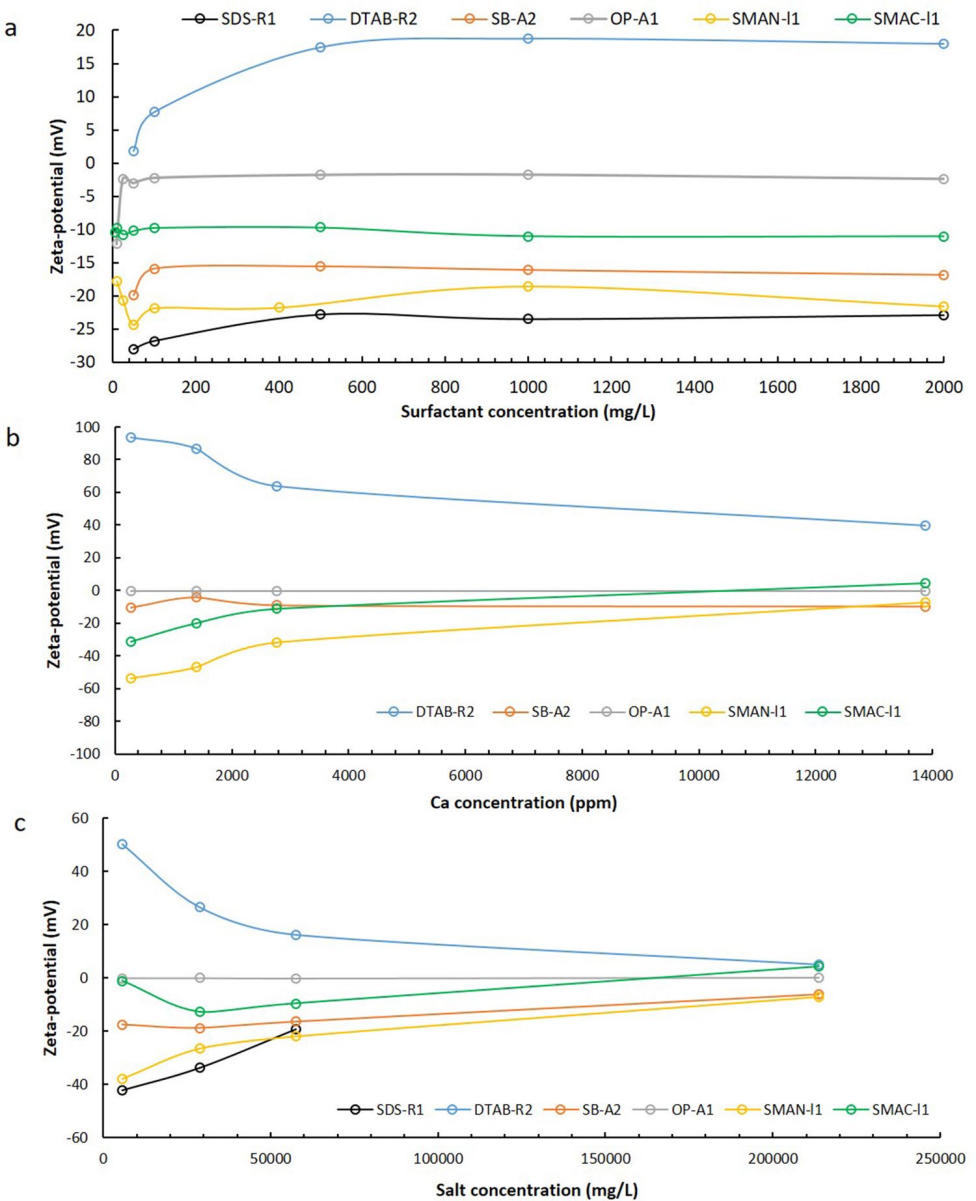
The zeta-potentials of oil droplets (**a**) in different concentrations of surfactants in HSW, (**b**) in 2000mg/L surfactants in different concentrations of calcium and (**c**) in 2000 mg/L surfactants in diluted HSW, HSW and connate water. Black line SDS-R1, blue line DTAB-R2, orange line SB-A2, gray line OP-A1, yellow line SMAN-l1, green line SMAC-l1.

Figure 2a shows the zeta-potential testing results of oil droplets in various concentrations of surfactants. In 50–2000 mg/L SDS-R1 solution (Fig. 2a black line), the zeta-potentials of oil droplets were all below − 20 mV, more negative compared to the value of − 12.7 mV without surfactant, indicating that the SDS-R1 is a strong potential determining surfactant in a wide concentration range. In 50 mg/L cationic surfactant DTAB-R2 solutions (Fig. 2a blue line), the zeta-potential of oil droplets was positive at + 1.76 mV. With increasing DTAB-R2 concentration, the zeta-potential increased quickly and became stable at + 18 mV when DTAB-R2 concentration is more than 500 mg/L. The zeta-potential of oil droplets in SB-A2 in HSW increased from − 19.8 mV to around − 16 mV in the concentration range (Fig. 2a orange line). Different from the charge carrying surfactants, non-ionic surfactant OP-A1 showed a charge shelter effect on the zeta-potential of oil droplets. In 10 mg/L OP-A1 solution, the zeta-potential was − 12.2 mV, showing no difference from the case without any surfactant. When OP-A1 was higher than 25 mg/L, the zeta-potential quickly dropped to near zero (− 2.4 mV), and did not change with the increase of OP-A1 concentration (Fig. 2a grey line). Coincidently, for all the four surfactants, the lowest concentration exhibiting evident impact on the zeta-potential of oil droplets was higher than their CMC in HSW. The zeta potentials of oil droplets stopped increasing when the surfactant concentrations were higher, which was possibly due to the saturation adsorption of surfactants at the oil/water interface.

The zeta-potential in the SMAN-l1 solution was around − 21 mV in a wide concentration range from 10 to 2000 mg/L. A lowest value appeared at 50 mg/L, which was slightly higher than its CMC in HSW. SMAC-I1 provided a zeta-potential of around − 10 mV (green line). All the measured absolute zeta-potential values were smaller than that in anionic surfactant, indicating that the zeta-potential controlled by surfactant mixtures were affected by the synergetic interactions of all the components.

After the discussion on the effect of surfactant concentration on zeta-potential result, the impact of salinity on surface charge was investigated. Figure 2b presents the influence of divalent ion Ca^2+^ on the zeta-potential of oil droplets in five surfactants at 2000 mg/L. SDS-R1 was not included because it will precipitate in 100 mg/L Ca^2+^ solution. It shows the Ca^2+^ can make  the zeta-potentials of  surfactant mixtures increase. This is caused by the charge neutralization at low concentration and suppression of double electric layer at high calcium concentration. In non-ionic surfactant solutions, the zeta-potential is independent of the calcium concentration, which is around − 1 mV. It is interesting that in cationic surfactant solutions the zeta-potential is high at low calcium concentration and calcium suppresses the surface charge at high concentration. As Ca^2+^ is much smaller than DTAB-R2 molecules, it can attach on the surface of oil droplets with DTAB-R2 and obtain a high zeta-potential at low concentration. The zeta-potential will be at maximum value until the Ca^2+^ cannot adsorb on oil droplets anymore because of static repulsion. Then with the increase of Ca^2+^ concentration, the suppression of double electric layer caused by the increase of ionic strength in solution will play a dominant role and decrease the zeta-potential value.

Figure 2c shows the results of the zeta-potential of oil droplets for six surfactants at 2000 mg/L in brines with varied salinities, including the brines with 10% and 50% of HSW, HSW and connate water. SDS-R1 in connate water is not included because of the precipitate problem. The results demonstrate that the absolute value of zeta-potential of oil droplets gradually decreases to a low value in very high salinity solution (connate water), except that the zeta-potential in the non-ionic surfactant solution is kept at around − 1 mV. It is interesting that the zeta-potential in SMAC-I1 solution turns to weakly positive in formation water, which might be attributed to the interactions between the surfactant and the cationic component in brine.

**Figure 3:**
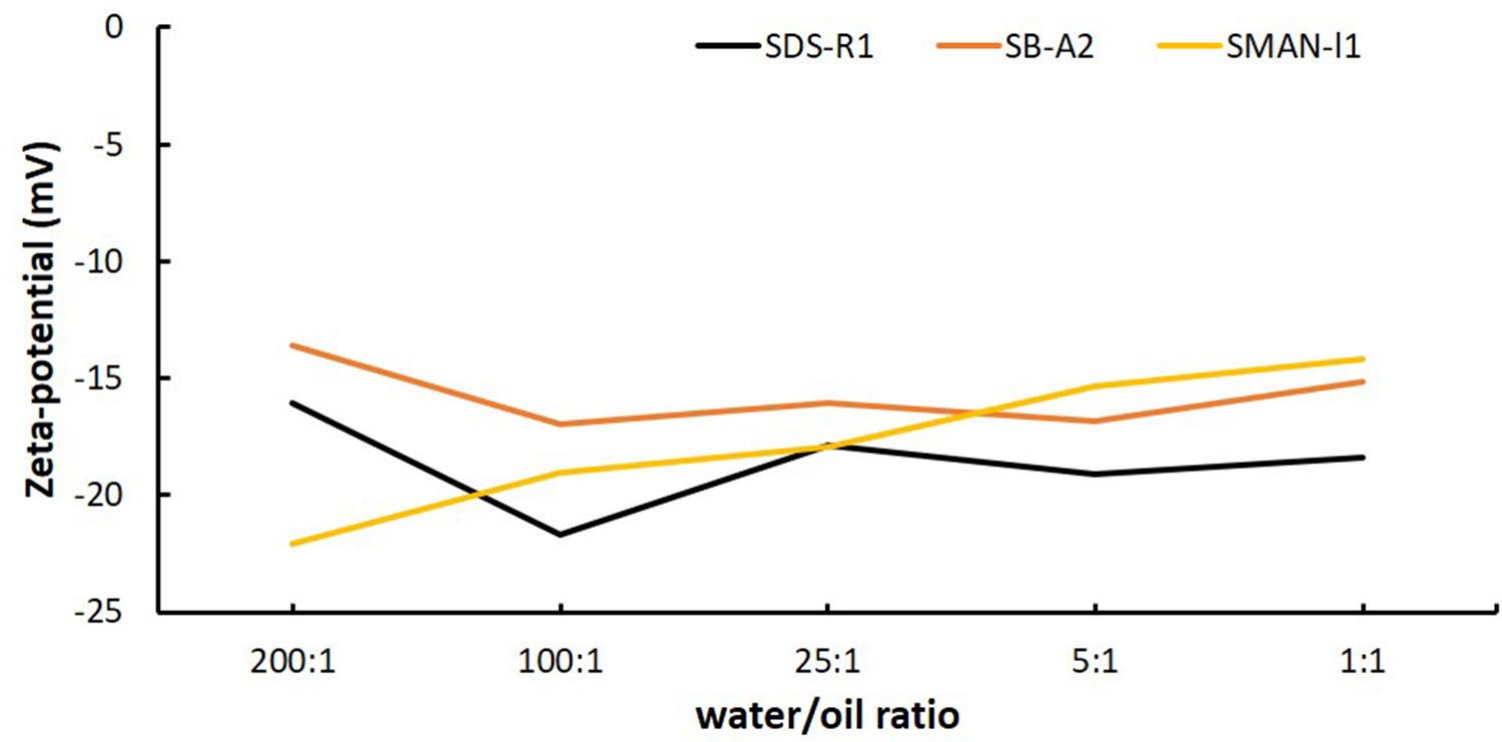
The zeta-potential of oil droplets in (**a**) SDS-R1 (black line), (**b**) SB-A2 (orange line) and (**c**) SMAN-I1 (yellow line) solutions with different water/oil ratio.

As the water/oil ratio is an important factor in the interactions between surfactant and oil, the zeta-potentials at different water/oil ratios in three surfactant (SDS-R1, SB-A2, SMAN-I1) in HSW were investigated (Fig. 3). The oil/water emulsion in bottom phase was collected. Although the zeta-potentials fluctuate slightly in SDS-R1 and SB-A2 solutions when the water /oil ratio decreases from 200:1 to 1:1, it is generally independent of the water/oil ratio. The zeta-potential of oil droplets in SMAN-I1 solutions increases from − 22.1 to − 14.2 mV when the volume of oil increases. This experiment indicates that the polarity of oil in water phase with surfactants is not obviously influenced by the water/oil ratio.

The zeta-potential measurements of oil droplets demonstrate that the surfactant type plays a dominant role in the surface charge of oil droplets in low salinity water. The anionic and zwitterionic surfactants make a negative surface charge, while the cationic surfactant makes a positive charge on oil droplets. Different from ionic surfactant, the non-ionic surfactant tends to reduce the zeta-potential of oil droplets. In surfactant mixtures, the positive charge and negative charge neutralize each other, and the overall zeta-potential of oil droplets is negative. In high salinity condition, the absolute values of zeta-potential are close to zero, and the polarities are affected by both the ions in brine and the surfactant types.

### Zeta-potentials of rock particles

**Figure 4:**
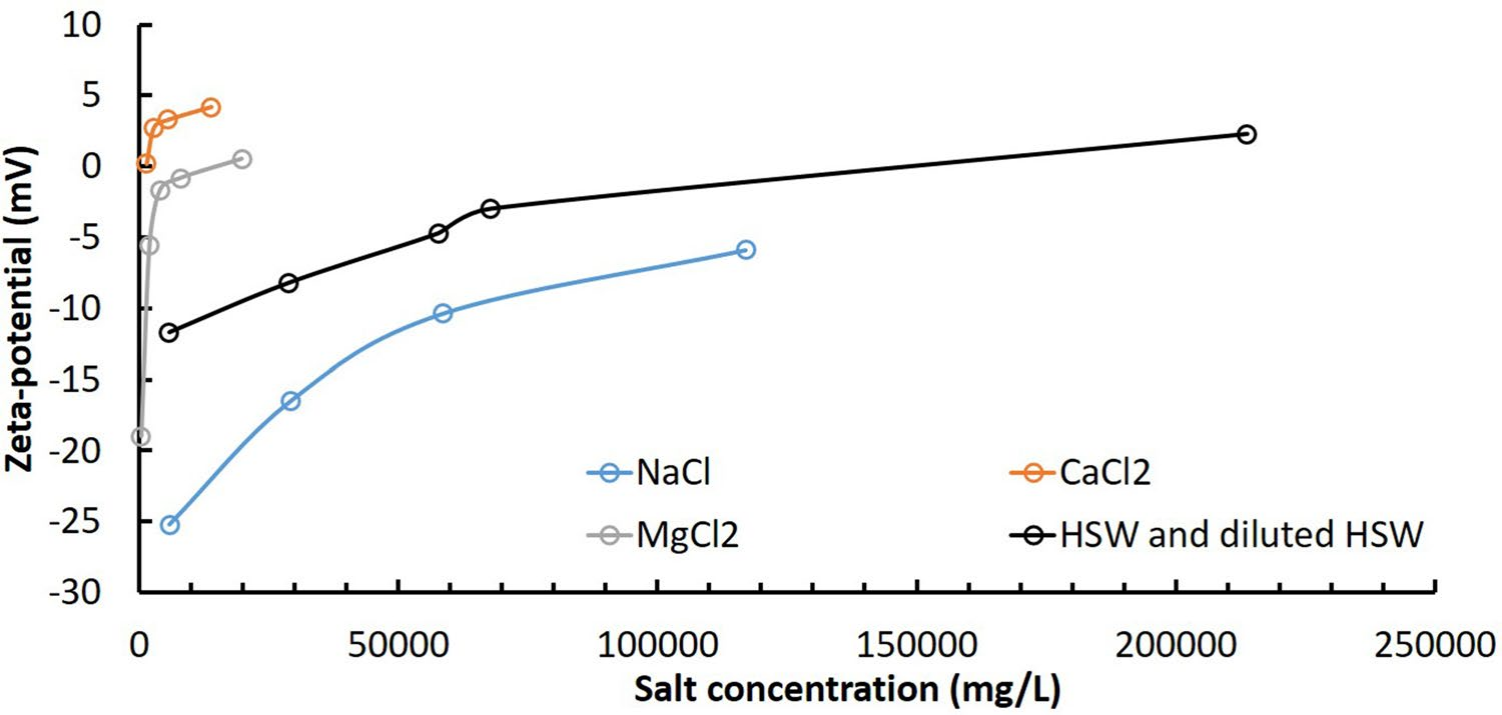
Salt effects on the zeta-potential of calcite particles.

Multivalent cations, such as Ca^2+^ and Mg^2+^, play an important role on the change of the surface charge of carbonate rock. Figure 4 presents the results of the surface charges of crushed core powders in four different brines, including NaCl, CaCl_2_, MgCl_2_ and HSW. In NaCl solution, the zeta-potential increases with increasing Na^+^ concentration, which is expected as the high concentration of Na^+^ suppresses the double electric layer, and decreases the absolute value of zeta-potential. But in CaCl_2_ solution, the zeta-potential of calcite particles turns to positive at low calcium concentration. This is because Ca^2+^ is one of the potential determining ions (PDI). The adsorption and ion exchange reaction of Ca^2+^ onto the particle surface lead to the changes of negative zeta-potential to positive values^29^. Mg^2+^ is another PDI for carbonate rock. When the concentration of Mg^2+^ ranges from 100 to 5000 mg/L, the trend line of zeta-potentials is similar to that with the addition of Ca^2+^. But the influence of Mg^2+^ on the zeta-potential is weaker than that of Ca^2+^. The measurement of zeta-potential in HSW is the overall effects of Na^+^ and Ca^2+^, and the value changes from negative to positive at high salinity condition (Fig. 4 black line).

**Figure 5:**
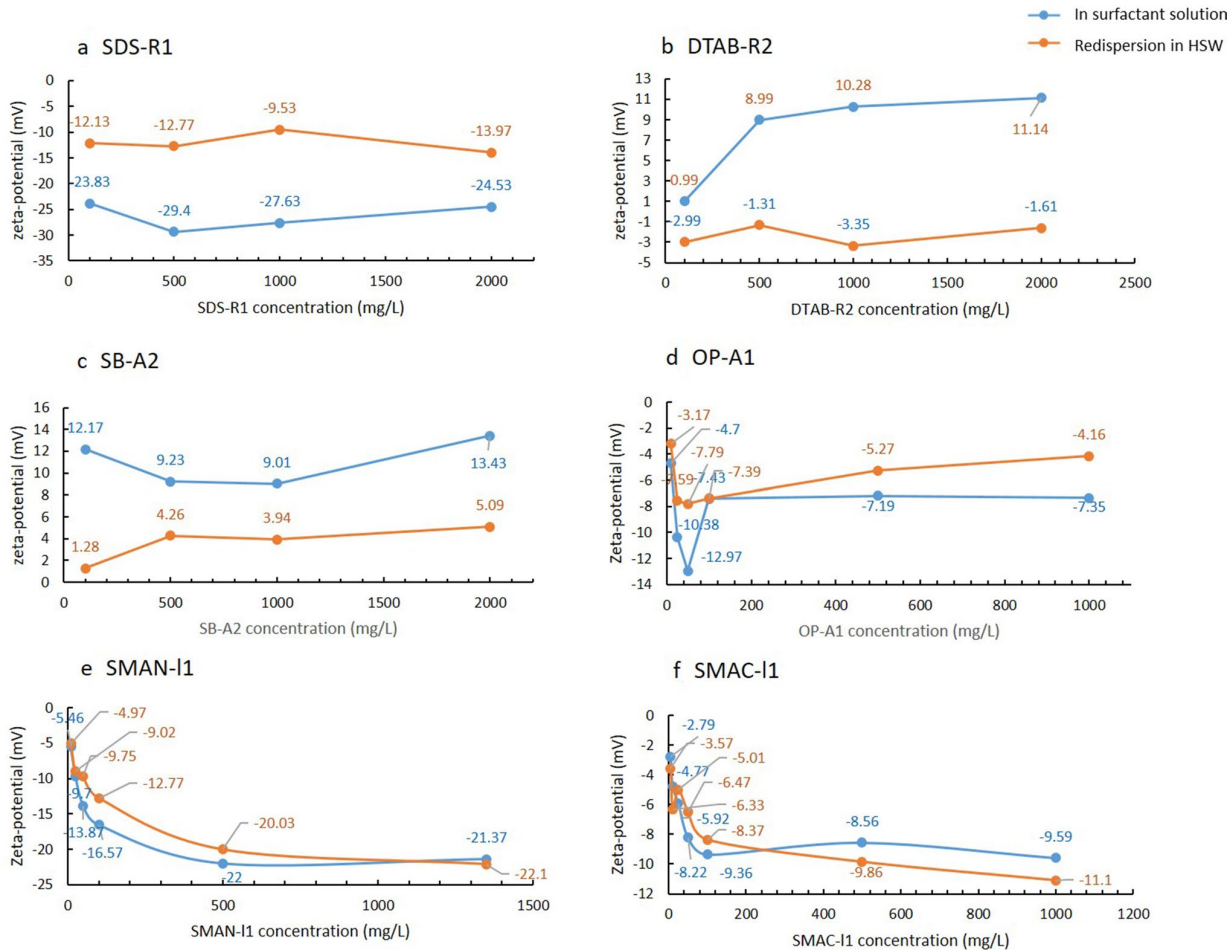
The zeta-potential of calcite particles in the presence of different concentration of surfactants (blue line) and after the surfactant solution is excluded and re-dispersed in HSW (orange line).

The influences of surfactant type on zeta-potential of calcite particles are evaluated in HSW. Figure 5 presents the results of zeta-potential as functions of surfactant concentration for six different surfactant solutions prepared by HSW. The zeta-potential of calcite particle in the absence of surfactant is − 4.72 mV (Fig. 4). The lowest zeta-potential value of − 29.4 mV is observed when using 500 mg/L SDS-R1 (Fig. 5a). At 2000 mg/L SDS-R1, the value increases to − 24.5 mV. This indicates that the SDS-R1 has the strong potential determining ability on rock particles. In 100 mg/L cationic surfactant (DTAB-R2) solutions, the zeta-potential of calcite particle shows a positive value of + 0.99 mV. As the DTAB-R2 concentration increases, the potential increases, and the increase is faster when the DTAB-R2 concentration is below 500 mg/L. At 2000 mg/L, it increases to 11.1 mV (Fig. 5b). It is interesting that the zwitterionic surfactant SB-A2 results in a positive zeta-potential of calcite particle, which is opposite to the zeta-potential of oil droplets. The zeta-potential of calcite particle is around + 11.0 mV in the test concentration range (Fig. 5c). The zeta-potential in OP-A1 is slightly negative, and a lowest value appears at around the CMC. For the surfactant mixtures of SMAN-I1 and SMAC-l1, the anionic component plays a dominant role, and the zeta-potential is negative in these two surfactants. The zeta-potential values become stable when the surfactant concentrations are higher than 500 mg/L (Fig. 5e, f).

The surface charge of calcite particles affected by surfactant is contributed to the adsorption of surfactant on rock surface in brines. To fully look into the surfactant influences on the surface charge of rock particle, zeta-potentials were measured in high salinity water after surfactants were excluded. After interacting with surfactant solution for one day at room temperature, the particle suspensions were centrifuge at 5000 G/min for 30 min. The supernatant with surfactant was removed, and the collected particles were re-dispersed in HSW. The orange line in Fig. 5 presents the zeta-potential of calcite particles after re-dispersion. In the case of SDS-R1, the zeta-potential is around − 12.1 mV. The absolute value is lower than that in the presence of surfactant, but is still higher than that not interacted with the surfactant. The reduction of zeta-potential of calcite particles is an indication of the strong adsorption of SDS-R1 on calcite surface. As to the cationic surfactant DTAB-R2, the zeta-potential after the exclusion of surfactant is − 2.3 mV, very close to the initial value of − 4.72 mV. This indicates that the interaction of DTAB-R2 and calcite surface is weak. The interaction of SB-A2 with carbonate rock is between the anionic surfactant and cationic surfactant. The residual zeta-potential is + 3.6 mV, presenting a limited long-term effect on the surface charge of calcite. The static adsorption experiments were conducted on the three surfactants. The adsorption amounts of SDS-R1, DTAB-R2, SB-A2 on calcite particles at room temperature were determined as 7.1 mg/g, 0.58 mg/g and 0.57 mg/g, respectively. The anionic surfactant has a higher adsorption value than cationic surfactant and zwitterionic surfactant because of the electrostatic forces. The results confirm the much stronger interaction of anionic surfactant with the calcite, which are consistent with the zeta-potential measurements. Besides, combining with the zeta-potential results of the particles after re-dispersion in HSW, it is assumed the desorption of SB-A2 is less than DTAB-R2 possibly because of the interaction of anionic groups with the particles. The non-ionic surfactant OP-A1 also presents a prolonged influence on the zeta-potential. Similar to SDS-R1, both surfactant mixtures SMAN-I1 and SMAC-I1 are strongly interacted with calcite surface. The residual zeta-potential of the particles are almost the same as in the presence of surfactants.

The results of zeta-potential measurements show that the surface charge property of oil droplets and rock particles is mainly controlled by the surfactant type, rather than the brine salinity or the ion components in brine. The anionic and cationic surfactants lead to the same surface charge of both rock particles and oil droplets. Zwitterionic surfactant makes a positive charge on carbonate rock particles, and a negative charge on oil droplets. Non-ionic surfactant plays a shelter role on the charged oil/water emulsions and rock particles, and the emulsions carry a weak negative charge in the non-ionic surfactant solution.

### Displacing experiment in carbonate micromodel

The two-dimensional (2D) micromodels fabricated with transparent windows are ideal tools for visual observation of pore scale flow at high temperature^30–33^. The current work uses the micromodels to understand the oil-water-rock interactions and flow behaviors in underground oil reservoirs. Most of the commercial micromodels are made of glass, silicon, and polymeric materials^34^. They have limitations in simulating the oil production process in carbonate reservoirs. The surface charge properties of these glass micromodels are different from the property of calcite. Therefore, we coat a CaCO_3_ nanocrystal layer onto the flow channels of glass micromodels, and fully convert the inner surface from silica to CaCO_3_^35^. A glass slide was treated by a similar CaCO_3_ coating process. The SEM images of the glass slide showed the CaCO_3_ coating was several micrometers thick (Figure S3). The contact angle measurement of oil droplets on the surface in HSW is at 62 °C.

Interfacial tension reduction and wettability alteration are two main factors to improve oil production by surfactant flooding. To present the influences from surface charge, the differently charged surfactants are grouped in two categories according to IFT values (in Table 1). One includes SDS-R1, DTAB-R2 and SB-A2 with IFT in the range of 10^−1^ mN/m. The other includes SMAN-I1 and SMAC-I1 with IFT in the range of 10^−3^ mN/m. Besides, the contact angle of oil droplets on CaCO_3_ coated surface in SDS-R1, DTAB-R2 and SB-A2 were determined as 110.9 ± 2.3°, 120.1 ± 1.2° and 115.0 ± 6.7°, respectively. The results show the wettability alteration effect can be negligible when using these surfactants.

**Table 1:** Summary of oil production by micromodel displacement tests.

Surfactant name	Surfactant type	Zeta-potential ζ_oil_/mV	Zeta-potential ζ_roc_/mV	Absolute zeta-potential value ζ_oil _+ ζ_rock_/mV	Incremental oil production/%	IFT (25°C)/mN/m
SDS-R1	Anionic	− 23	− 25	− 48	21.0	0.46
DTAB-R2	Cationic	+ 18	+ 11	+ 29	15.5	0.67
SB-A2	Zwitterionic	− 17	+ 13	4	12.6	0.44
SMAN-I1	Anionic/nonionic mixture	− 17	− 17	− 34	39.4	0.001
SMAC-I1	Anionic/cationic mixture	− 11	− 11	− 22	30.6	0.0009

**Figure 6:**
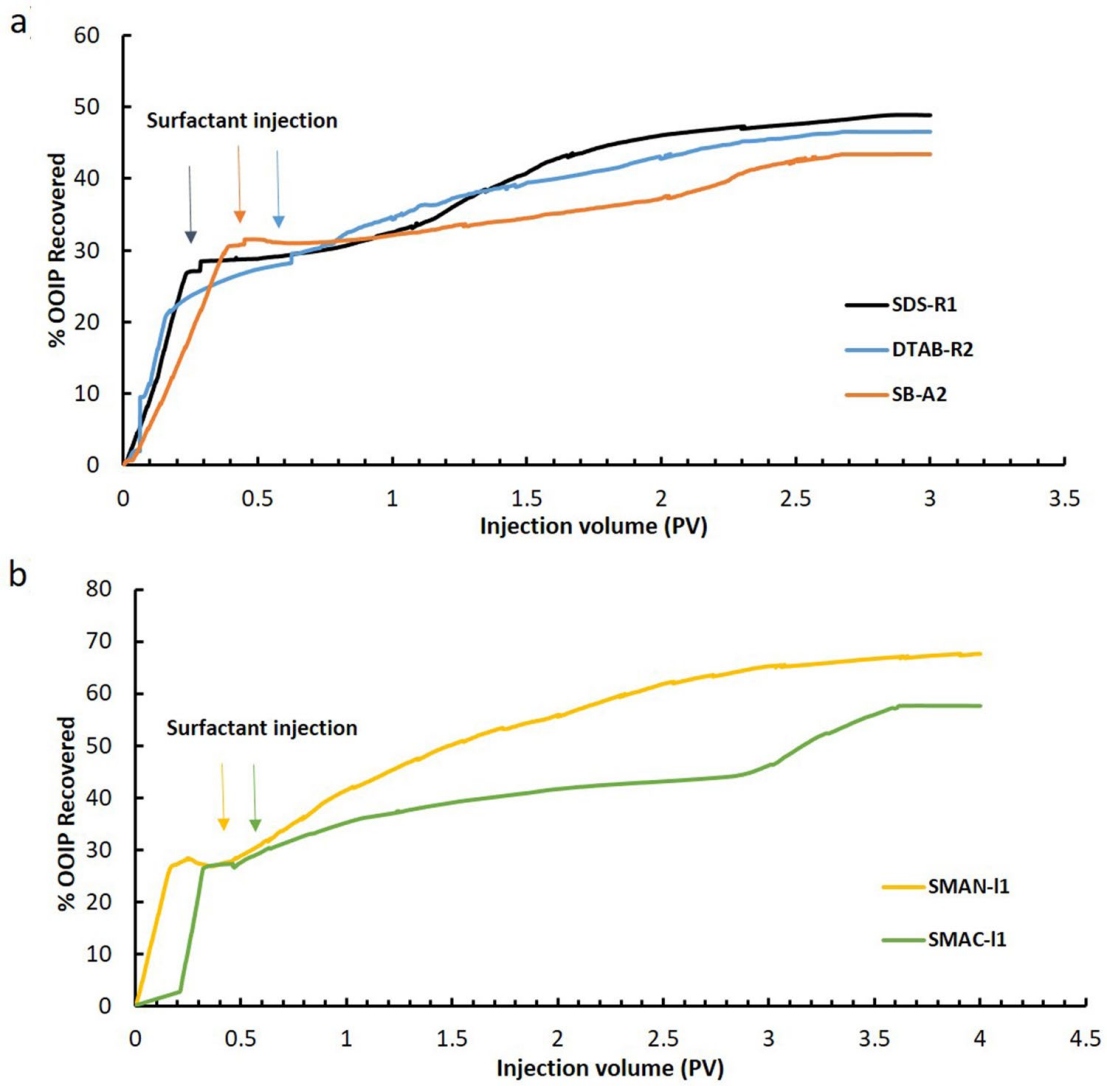
Oil production rate by injecting differently charged surfactants. (**a**) The injection of surfactants with IFT around 10^−1^ mN/m. SDS-R1 in black line, DTAB-R2 in blue line and SB-A2 in orange line; (**b**) the injection of surfactants with IFT around 10^−3^ mN/m. SMAN-I1 in yellow line, SMAC-I1 in green line.

Figure 6 presents the oil production rate versus injected pore volumes of brines and surfactant solutions. It shows that the highest oil production rate is achieved when anionic surfactant SDS-R1 solution was injected, while the lowest oil production rate occurs when using zwitterionic surfactant SB-A2 (Fig. 6a). The lower oil production of SB-A2 flooding is possibly caused by the static interaction between the oil droplets and rock. From the absolute value of zeta-potential ζ_oil _+ ζ_rock_ and the oil production rate in Table 1, it is also found that the higher oil production rate by SDS-R1 injection than that by DTAB-R2 injection was likely owing to the higher absolute zeta-potential value of ζ_oil _+ ζ_rock_.

Figure 6b presents oil production rate by injection of SMAN-l1 and SMAC-l1 when the IFTs are at 10^−3^ mN/m, respectively. The incremental oil recovery of SMAN-l1 injection is 39.4%, which is higher than that of SMAC-I1 injection of 30.6%. Besides, the absolute zeta-potential value of ζ_oil _+ ζ_rock_ of SMAN-l1 is 34mV, higher than that of SMAC-l1 of 22 mV. The results indicate that the bigger absolute zeta-potential value of ζ_oil _+ ζ_rock_ resulted in the higher oil production rate. Though IFT plays a dominant role in oil production by comparing Fig. 6a, b, it is found the surface charge can obviously affect oil recovery at the same IFT range. And stronger static repulsion between oil droplets and rock surface could improve oil production rate. The diameters of emulsions stabilized by these surfactants are in the range of several micrometers (Figure S4 and Table S2), which supported that these oil droplets could be affected by the static forces.

### Mechanism of surfactant charge effects

Theoretical works have been conducted on the simulation and microscopic description of physical and chemical processes in charged porous media, as well as Darcy-scale models to simulate the behavior of these systems at larger physical scales^36–38^. Rabbani et al. reported numerical simulations of two-phase immiscible fluids displacement to reveal the wettability effects in this process^39^. Although the oil/rock interaction in the detachment process has been intensely studied, little experiment researches are reported on the surface charge effects on the transportation of oil droplets in the charge rock media^40^.

**Figure 7:**
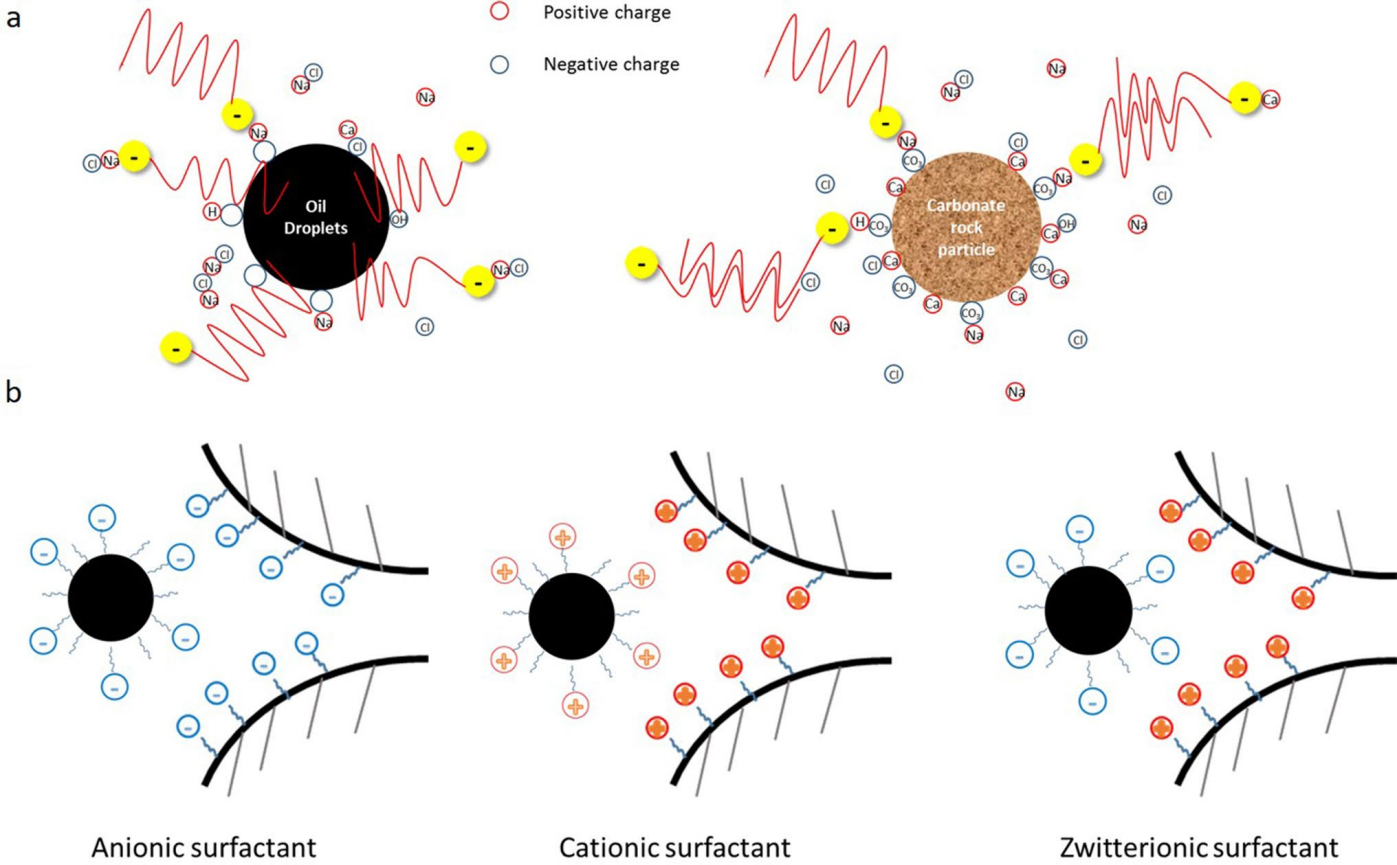
(**a**) The scheme of zeta-potential of oil droplets (left) and carbonate rock particles (right) in anionic surfactant and brine solutions; (**b**) the scheme transportation of charge oil droplets in charged porous media.

In this study, it is emphasized that the transportation of oil in porous media is affected by the charged oil droplets and rock surfaces. In surfactant flooding, the surface charge of oil droplets and carbonate rock are determined by the charge state of surfactants and salt compositions (Fig. 7a). Figure 7b presents some of the possible scenarios when a charged oil droplet passes through a charged pore throat. It indicates that the transportation of charged oil/water emulsion in the porous media is improved when the signs of the surface charges are the same, and the oppositely charged emulsions and rock surfaces will impede the transportation of oil.

## Conclusions

In this work, we investigated the surface charges of oil droplets and carbonate rock surfaces induced by the addition of surfactants. We looked into the surface charge impact on oil production enhancement in pore porous media, rather than the effects of interfacial tension and wettability alteration. The following conclusions were drawn based on the experimental results.

1. High concentrations of Na^+^ and Ca^2+^ decrease the absolute value of the zeta-potential of both oil droplets and rock particles.
2. The anionic and cationic surfactants lead to the same surface charge for both carbonate rock particles and oil droplets, while the zwitterionic surfactant makes a positive charge on carbonate rock particles, and a negative charge on oil droplets.
3. Micromodel displacement tests show that the anionic surfactant flooding achieves the highest oil production rate, while the zwitterionic surfactant flooding shows the lowest value. This indicates that the same surface charge may improve the transportation of charged oil/water emulsion in the porous media, resulting in a higher oil production rate than the oppositely charged oil droplets and rock particles.

## Methods

### Materials

#### Bine and oil

The salts used for preparation of high salinity water (HSW) and other brines were NaCl, CaCl_2_, MgCl_2_, Na_2_SO_4_, and Na_2_CO_3_ from Sinopharm Reagent (Beijing). The densities of the HSW were 1.036 g/cm^3^ at 30 °C. The HSW composition is Na^+^: 18,300 mg/L; Ca^2+^: 650 mg/L; Mg^2+^: 2110 mg/L; SO_4_^2−^: 4290 mg/L; Cl^−^: 32,200 mg/L; HCO_3_^−^: 120 mg/L. And the total dissolved solid (TDS) was 57,670 mg/L. Crude oil from carbonate reservoirs was used (density = 0.882 g/cm^3^ at 25°C).

#### Surfactants

DTAB-R2 (quaternary ammonium cationic surfactant), SDS-R1 (sulfate anionic surfactant), SB-A2 (zwitterionic surfactant and OP-A1 (non-ionic surfactant) were from Sinopharm (Beijing, China) and the chemical structures are given in Figure S2. SMAN-I1 was a mixture of α-olefin sulfonate/glucoside (C12–C16). SMAC-I1 was a mixture of CTAB and a sulfonate surfactant with ethylene oxide (EO) and propylene oxide (PO) groups.

#### Other chemicals

NaOH, chloroform, ammonium hydroxide (water solution), toluene and hydrogen peroxide (30% in water) were purchased from Sinopharm Reagent (Beijing). Silane coupling reagent (40% in water) and methanol were from Sigma-Aldrich. Pure water used in the experiments was from a Thermo water purified system.

### Zeta-potential measurement of emulsions and rock particles

Zeta-potential measurements were performed on ZetaSizer nano series - Nano-ZS (Malvern Instruments Ltd., United Kingdom). The instrument measures the direction and velocity of a particle in an applied electrical field via phase analysis light scattering and laser Doppler velocimetry. The calculated electrophoretic mobility is converted into zeta potential values using the Smoluchowski model. Before measuring the rock particles or emulsions in surfactants solutions, the zeta-potential of surfactant solutions was determined to make sure the results of rock particles or emulsions will not be interfered by surfactants aggregates.

#### Rock particles

The rock particles were crushed to less than 35 µm in diameter. The suspensions were prepared by mixing 0.1 g of milled-carbonate particles with 10 g of brine, which represents 1 wt% of the aqueous solution. The mixtures were sonicated for 20 min. The pH of the resultant solutions was around 7. The average value of three measurements with 15–100 runs each was selected as the expected zeta-potential.

#### Emulsions

0.1 mL crude oil was dropped into 20 mL solutions. The solution was mixed using IKA homogenerator at 5000 rpm for 5 min to make an emulsion solution. The emulsion in the middle of the solution was taken out for zeta-potential analysis without further treatment. The average value of three measurements with 15–100 runs each was selected as the zeta-potential.

### Contact angle measurement

The contact angle measurements were performed on the optical contact angle measuring system, OCA 15, made by Dataphysics (Germany). A 0.5 cm×0.5 cm piece of CaCO_3_ glass slide was immersed in HSW or surfactant solutions. Then the contact angle of the oil droplet attached on the bottom of the surface was calculated and recorded automatically based on the captured image by camera. Each measurement lasts for more than half an hour until the contact angle did not change any more. And the measurements were conducted three times at different spots for each surfactant.

### Static adsorption on carbonate rock

The carbonate core plug was crushed and grinded. The particles of 177–250 µm in size were used in the static adsorption experiment. Then 5 mL 2000 mg/L surfactant solutions with 1 g rock particles were incubated at 25 °C for 24 h. After the solutions were separated by centrifugation at 5000 rpm for 30 min, the surfactant concentration in the supernatant was determined by total carbon organic analyzer (Shimadzu, TOC-L, Japan) to calculate the adsorption amount.

### Fabrication of carbonate micromodel

The pattern of the micromodel slice was based on the image of micro-channel from a carbonate core plug with the size of flow channel at ~ 100 µm. The fabrication of carbonate micromodel includes three steps, (1) micromodel cleaning by injection of piranha solution, (2) seed growth by injecting the silane coupling reagent into the microchannels followed by injection of 0.05 mol/L CaCl_2_, and (3) CaCO_3_ growth by alternatively injecting 0.05 mol/L CaCl_2_ and 0.05 mol/L Na_2_CO_3_ into the micro-channels. The micromodel was cleaned by injection of water after fabrication and dried at 60 °C for further use. The details of the fabrication method are in Supplementary Information and referred to the Reference^35^.

### Micromodel displacing test

The micromodel apparatus is shown in Figure S5 (Supplementary Information). Before the oil displacement test, the flow channel was rinsed in connate water in the vacuum dryer. The vacuum was kept for 30 min using a vacuum pump to make sure the air dissolved in connate water was removed. After the micromodel was saturated by connate water, it was connected in a displacing system with inlet connected to surfactant flooding tubes and bypass, and outlet connected to oil injection tubes and bypass. To saturate the micro channels with oil, the oil was injected from the outlet and the inlet was connected to the bypass of waste at 0.5 mL/min. Surfactant solution or HSW was injected from the inlet of the micromodel and the outlet is connected to bypass. The injection scheme is HSW injection followed by surfactant flooding. The injection rate is both set at 2.5 µL/min. The experiments ended until the oil was not moved. All the experiments were repeated at least twice. After the oil displacement experiment, the micromodel was washed by water, methanol and toluene. The wash flush was repeated three time and the micromodel was dried at 60 °C before the next experiment. The carbonate layers on the microfluidic channel was removed using chromic acid solution and re-fabricated after 10 times flooding experiments.

## Supplementary Information

Supplementary Information.
