# Supplementary material for: Manipulation of surface charges of oil droplets and carbonate rocks to improve oil recovery
Source: Sci Rep. 2021 Jul 15;11:14518. doi: 10.1038/s41598-021-93920-3 (PMC8282872; doi:10.1038/s41598-021-93920-3)
Supplement: Supplementary file 1 — Supplementary Information. [file 41598_2021_93920_MOESM1_ESM.pdf]

Supplementary Information

Experimental

CMC measurement

The measurement of surface tension of surfactant solution was conducted by plate method using DCAT21 surface tensiometer (Dataphysics, Germany). The critical micelle concentration was obtained with surface tension at different concentrations through an auto-dilution program at room temperature.

SEM images of the coated glass slide

A glass slide was coated by $CaCO_3$ using the same procedure as the preparation of glass micromodels. Before SEM tests, the glass slide was coated by Pt to improve the electronconductibility. The images were recorded in HITACHI SU8000 cold field emission scanning electron microscope.

Measurement of emulsion size

The measurement of emulsion size was performed on Olympus Microscopy BX61. The emulsions were prepared by adding 0.2 mL crude oil in 10 mL surfactant solution and mixing using IKA homogenerator at 5000 rpm for 5 mins. 10µL solution was dropped on a microscopy slide and observed. The average diameter and diameter distribution of obtained emulsion images were process using MATLAB software (2019a).

Fabrication of carbonate micromodel

Micromodel cleaning. Pump the base piranha solution (3:1 ammonium hydroxide to hydrogen peroxide) in the channels at 0.1 mL/min for 3 mL, and keep the channel at room temperature for half hour. Clean the residual solution in the channels by air blow. 1M NaOH aqueous solutions was pumped through the microfluidic channels at 0.1 mL/min for 30 mins. The micromodel was then rinsed in DI water and residual water was removed by air blow.

Seed growth. 2 mL of silane coupling reagent (40% in water) was mixed with 30 mL chloroform-water (1:1) under magnetic stirring. Adjust the pH to 1.5 using 20 droplets hydrochloric acid. The solutions were stirred for 30 mins and the silane reagent will be in chloroform phase (bottom). Separate the chloroform phase and water phase in separation funnel. Pump the chloroform phase through the micromodel at 0.1 mL/min for 5 mins. Keep for 15 mins before clean the micromodel by air flow. Repeat pump chloroform solution for 3-5 times. Rinse the micromodel in ethanol to remove chloroform. Inject 0.05 mol/L $CaCl_2$ in the microchannel manually and put the slide at 60^oC overnight.

$CaCO_3$ growth. 0.05 mol/L $CaCl_2$ was pumped through the channel at 0.1 mL/min for 2 mins, stay for 10 mins. The residual solution was removed by flow of air. Then pump 0.05 mol/L Na_2CO_3 through the channel at 0.1 mL/min for 2 mins, stay for 10 mins. Blow the channel with air. Alternate the injection of $CaCO_3$ and Na_2CO_3 process for 10 times. Rinse the channels by water and dried at 60^oC in air.

Results

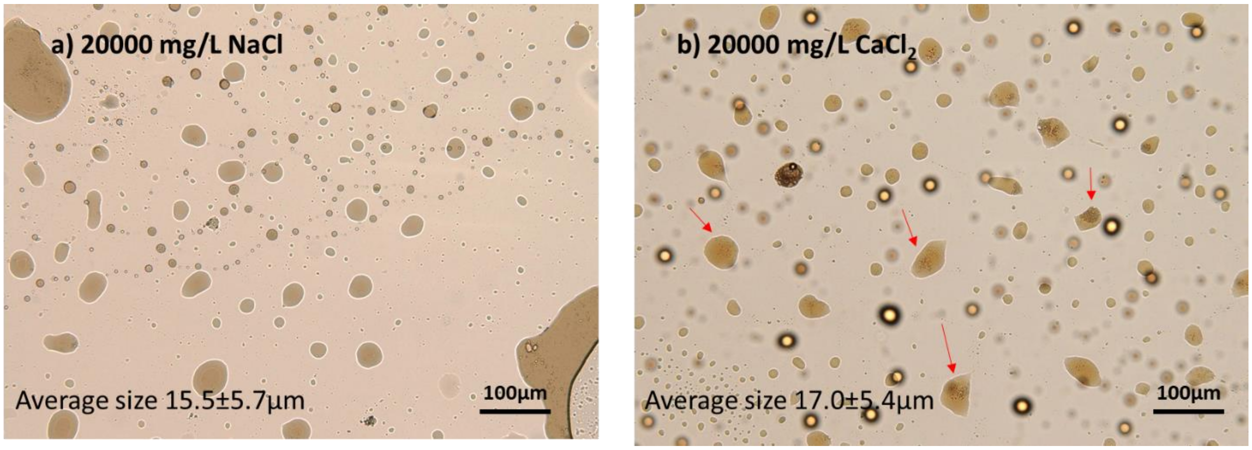

Figure S1. Microscopy images of the emulsions in a) 20000mg/L NaCl and b) 20000mg/L $CaCl_2$.

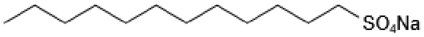

SDS-R1

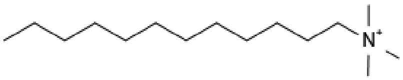

DTAB-R2

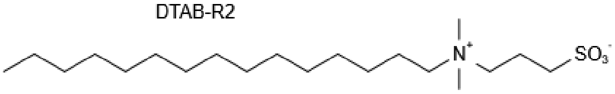

SB-A2

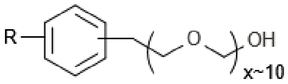

OP-A1

Figure S2. Chemical structures of SDS-R1, DTAB-R2, SB-A2 and OP-A1.

Table S1. Summary of critical micelle concentration (CMC) of surfactants in high salinity water.

**Surfactant Name**	SDS-R1	DTAB-R2	SB-A2	OP-A1	SMAN-I1	SMAC-I1
**Surfactant Type**	Anionic	Cationic	Zwitterionic	Non-ionic	Anionic/non-ionic mixture	Anionic/cationic mixture
**CMC (mg/L)**	35	45	50	16	22	12

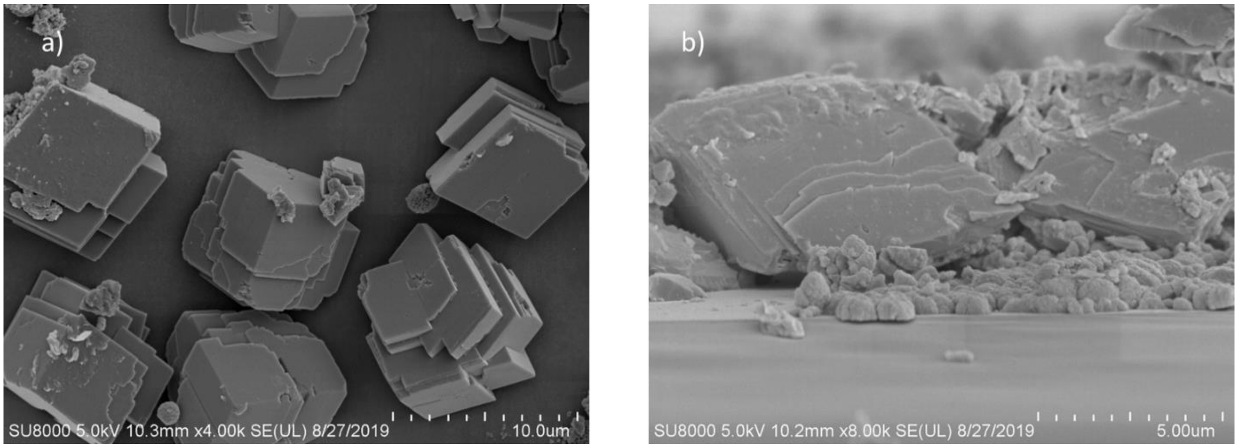

Figure S3. SEM images of the glass slide surface after modification carbonate nanocrystals. a) top views and b) side views.

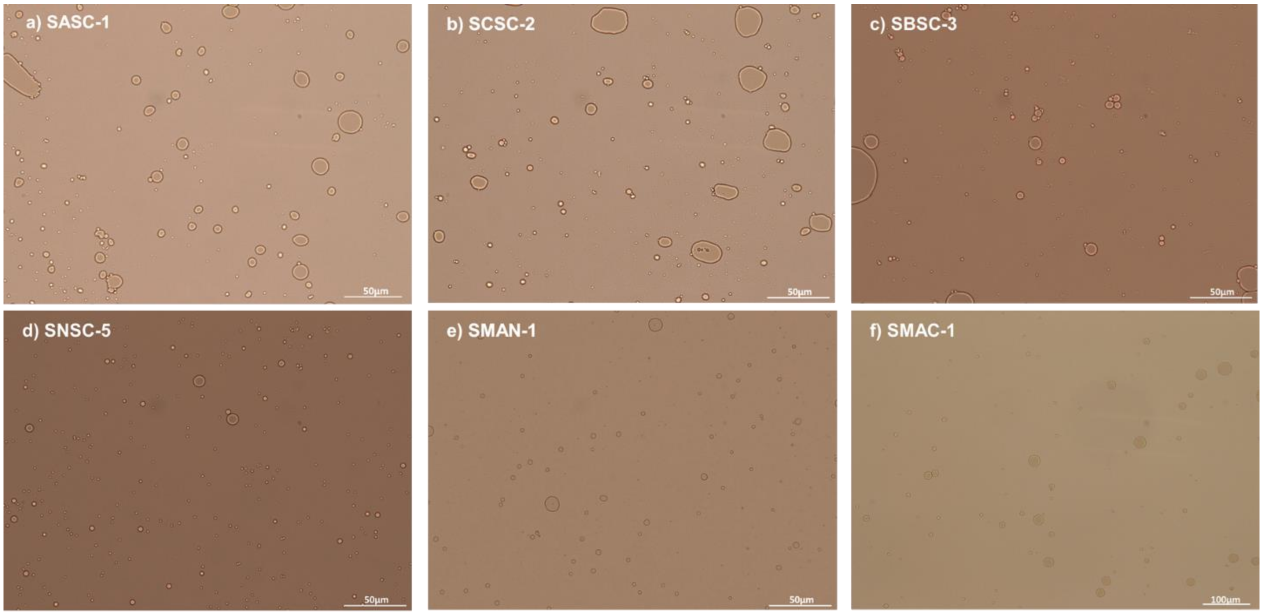

Figure S4. Microscopy images of the emulsions stabilized by a) SASC-1, b) SCSC-2, c) SBSC-3, d) SNSC-5, e) SMAN-1 and f) SMAC-1.

Table S2. Emulsions size stabilized by the six surfactants

Name	SDS-R1	DTAB-R2	SB-A2	OP-A1	SMAN-I1	SMAC-I1
Size (μm)	2.28±0.03	5.26 ±0.09	5.86±0.04	1.02 ±0.05	1.35±0.03	4.66±0.66

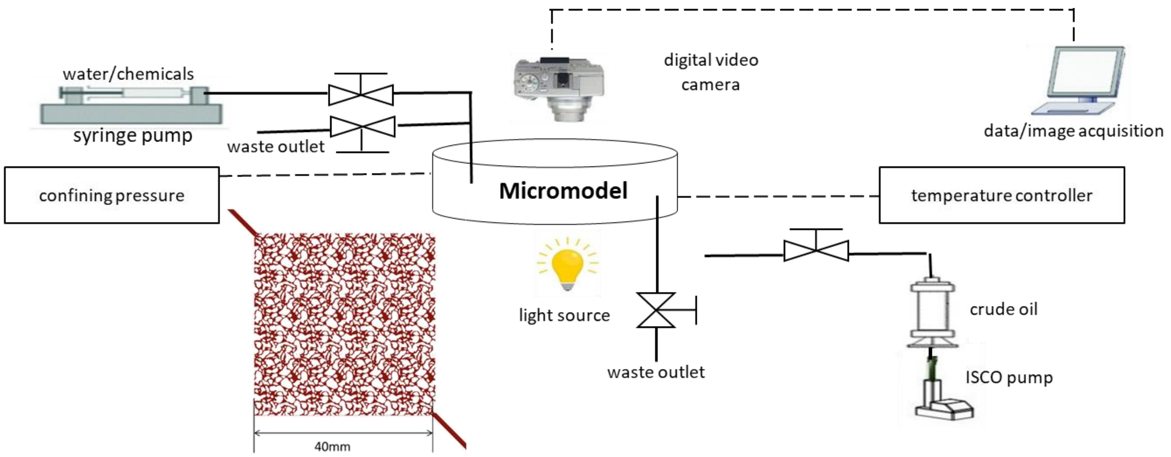

Figure S5. Micromodel displacement setup.
